# Chickens Expressing IFIT5 Ameliorate Clinical Outcome and Pathology of Highly Pathogenic Avian Influenza and Velogenic Newcastle Disease Viruses

**DOI:** 10.3389/fimmu.2018.02025

**Published:** 2018-09-14

**Authors:** Mohammed A. Rohaim, Diwakar Santhakumar, Rania F. El Naggar, Munir Iqbal, Hussein A. Hussein, Muhammad Munir

**Affiliations:** ^1^Department of Virology, Faculty of Veterinary Medicine, Cairo University, Giza, Egypt; ^2^The Pirbright Institute, Woking, United Kingdom; ^3^Division of Biomedical and Life Sciences, Faculty of Health and Medicine, Lancaster University, Lancaster, United Kingdom; ^4^Department of Virology, Faculty of Veterinary Medicine, University of Sadat City, Sadat, Egypt

**Keywords:** innate immunity, antiviral response, transgenic, viruses, host registance

## Abstract

Innate antiviral immunity establishes first line of defense against invading pathogens through sensing their molecular structures such as viral RNA. This antiviral potential of innate immunity is mainly attributed to a myriad of IFN-stimulated genes (ISGs). Amongst well-characterized ISGs, we have previously shown that antiviral potential of chicken IFN-induced proteins with tetratricopeptides repeats 5 (chIFIT5) is determined by its interaction potential with 5′ppp containing viral RNA. Here, we generated transgenic chickens using avian sarcoma-leukosis virus (RCAS)-based gene transfer system that constitutively and stably express chIFIT5. The transgenic chickens infected with clinical dose (EID_50_ 10^4^ for HPAIV and 10^5^ EID_50_ for vNDV) of high pathogenicity avian influenza virus (HPAIV; H5N1) or velogenic strain of Newcastle disease virus (vNDV; Genotype VII) showed marked resistance against infections. While transgenic chickens failed to sustain a lethal dose of these viruses (EID_50_ 10^5^ for HPAIV and 10^6^ EID_50_ for vNDV), a delayed and lower level of clinical disease and mortality, reduced virus shedding and tissue damage was observed compared to non-transgenic control chickens. These observations suggest that stable expression of chIFIT5 alone is potentially insufficient in providing sterile protection against these highly virulent viruses; however, it is sufficient to ameliorate the clinical outcome of these RNA viruses. These findings propose the potential of innate immune genes in conferring genetic resistance in chickens against highly pathogenic and zoonotic viral pathogens causing sever disease in both animals and humans.

## Introduction

Virus recognition by germ-line-encoded intracellular receptors underlines the potency of innate immune responses in restricting virus infections and disease progressions (1, 2). These intracellular receptors discriminate the host (self) nucleic acid from the virus (non-self) nucleic acid by the presence of molecular signatures in the viral genomic material. One of the well-characterized signatures of innate immune induction is the presence of triphosphate group (5′-ppp) in viral RNA which is absent in any RNA species of the host (3). Beside canonical cellular receptors including Toll-like receptors, retinoic-acid-inducible protein 1 (RIG-I)-like receptors, and nucleotide oligomerization domain-like receptors, recently a novel class of IFN-effectors, known as IFN-induced proteins with tetratricopeptides repeats (IFITs) which has been identified to directly engage with 5′-ppp viral RNA (4, 5).

Amongst viral RNA-recognizing cellular factors, IFITs proteins are the most transcribed and translated family of virus- and IFN-regulated proteins (6–8). These proteins are evolutionary conserved with at least four well-characterized paralogs in humans; IFIT1 (ISG56), IFIT2 (ISG54), IFIT3 (ISG60), and IFIT5 (also known as ISG58) (7–9). These IFNs and virus-responsive proteins are implicated in the regulation of several physiological (protein translation and cell proliferation) and pathological (inhibition of viruses) processes in mammals (7, 10). Among these functions, the implication of IFIT proteins in establishing host resistance against viruses has been well documented (4, 10, 11). Different biological, biochemical, and structural approaches have mapped the nature of RNA that is recognized by IFIT proteins including 5′-ppp, AU-rich elements, and initiator tRNAs (4, 9, 11, 12). Additional evidences suggested that IFIT5 proteins can perform their antiviral activities by two possible ways; sequestration of viral RNA translation and initiation of innate immune responses (9, 13). Both these activities augment the antiviral potential of IFIT5 against RNA viruses such as orthomyxoviruses (e.g., avian influenza viruses, AIV) and paramyxoviruses (e.g., Newcastle disease virus, NDV).

Highly pathogenic IAV (HPAIV) and velogenic NDV (vNDV) are causing devastating economical and welfare impacts on the poultry, and HPAIV are posing significant human health implications, around the globe (14, 15). Majority of negative sense single stranded RNA viruses, including HPIAV and vNDV, produce 5′-ppp containing RNA during the course of virus replication (16–18). Interaction of 5′ppp RNA and cellular receptors (e.g., IFIT5) leads to the induction of cytokines and may underline the antiviral state of the host with variable clinical or pathological outcomes. Understanding host factors that contribute in the pathobiology of viruses in their natural hosts may help to devise effective intervention strategies and to define the genetic markers of disease resistance. In the present study, we investigated the *in vivo* impact of chicken IFIT5 (chIFIT5) against HPAIV and vNDV-induced infections in transgenic chickens generated by the RCAS-based retroviral gene transfer system.

## Materials and methods

### Viruses and virus titration assays (EID_50_)

HPAIV H5N1 (strain A/chicken/Egypt_128s_2012) and vNDV (strain NDV-B7-RLQP-CH-EG-12 were kindly provided by Prof Hussein Ahmed (Virology Department, Faculty of Veterinary Medicine, Cairo University, Egypt). Both viruses were propagated in 9 days old specific pathogen free (SPF) chicken eggs. Infective allantoic fluid from the inoculated eggs was diluted in brain-heart infusion buffer (BHIB) and the median egg infectious doses 50 (EID_50_) were determined in SPF eggs using the Reed and Muench method (19).

### Construction of transgene expressing RCAS reverse genetic systems

The ORF of chicken IFIT5 was amplified from RNA extracted from the NDV-infected primary chicken embryo fibroblasts (CEFs), was sequence verified, codon optimized and chemically synthesized in-fusion with V5-tag, and sub-cloned into an improved version of RCASBP(A)-ΔF1 (kindly provided by Stephen H. Hughes, National Cancer Institute, MD, USA) via the *ClaI* and *MulI* restriction sites which replace the *src* gene while maintaining the splice accepter signals. The resultant constructs were named as RCASBP(A)-chIFIT5. Similarly, a GFP encoding RCASBP(A), referred as RCASBP(A)-eGFP, was generated by introducing the coding sequence of the GFP in between the *ClaI* and *MulI* sites. The inserted gene orientation and sequence validity were confirmed by DNA sequencing.

### Rescue of RCAS viruses and validation of transgene expression

To rescue recombinant RCASBP(A) viruses, a total of 2.5 × 10^5^ DF-1 cells were seeded in 25 cm^2^ flasks and maintained at 37°C, 5% (vol/vol) CO_2_ for 24 h (~80% confluent). Cells were washed with PBS and transfected with 2.5 μg of each of the RCASBP(A)-eGFP, and RCASBP(A)-chIFIT5 plasmids using Lipofectamine 2000 in OptiMEM with a pre-determined optimized ratio of 1:6 (Invitrogen, Carlsbad, CA, USA). Media were changed 6 h post transfection and the cells were maintained in DMEM supplemented with 10% FCS and 1% penicillin/streptomycin for 48 h. Expression of the reporter gene (GFP) was monitored using fluorescence microscopy whereas replication efficiencies of chIFIT5 expressing retroviruses were assessed by staining the structural protein of RCASBP(A) and V5 tag. The GFP/gag expression-confirmed cell cultures were split into 25 cm^2^ flasks and were passaged again into 75 cm^2^ flasks after 3 days. Finally, cells were expanded into 150 cm^2^ flasks until the desired number (10^6^ cells/egg) was achieved.

### Confocal microscopy

Chicken cells were transfected with individual or combined plasmids for indicated time points using Lipofectamine 2000 (Invitrogen) at a ratio of 1:3 or were infected with lentiviruses, retroviruses or NDV-GFP for indicated time points. These cells were then fixed for 1 h in 4% paraformaldehyde and permeabilised using 0.1% Triton-X100 before incubation with primary antibodies raised against V5 tag, or *gag* protein of retroviruses. Additionally, depending upon the experimental needs, different fluorescent markers (RFP, GFP) were used. Afterwards, cells were incubated with corresponding secondary antibodies at 37°C for 2 h. After brief staining with 4′, 6-diamidino-2-phenylindole (DAP1) (nuclear), slides were visualized using a Leica SP5 confocal laser-scanning microscope.

### Animals

SPF eggs were acquired from a local supplier in co-operation with Virology Department, Faculty of Veterinary Medicine, Cairo University, and the Central Lab for Evaluation of Veterinary Biologics Abbassia, Egypt. Transgenic chickens were generated as described below and each group of birds was housed separately at containment level 4 isolators. Food and water were provided *ad libitum*, and general animal care was provided by the animal house staff as required for each groups.

### Generation of transgenic chickens

Mosaic-transgenic chicken embryos were generated by inoculation of 1 million RCASBP(A)-chIFIT5-infected DF-1 cells or non-infectious DF-1 cells using 24G needs at day 2 post-embryonation in SPF chicken eggs. Embryos were fixed for 2 h post-inoculation before incubation at 37°C with 60–80% humidity in rotating incubator (twice daily). Transgenic embryos were allowed to hatch naturally at 21 days of incubation or were manually hatched on 22 days of embryonation.

### Challenge experimental design

#### Experiment 1: virus dose optimization

To effectively monitor the dose of viruses that can either induce clinical signs or mortality, a total of 10 SPF chicks (12 days old) were individual challenged with a dose of 10^4^, 10^5^, 10^6^ EID_50_ of HPAIV-H5N1. Similarly, vNDV was used to challenge 10 SPF chicks with a dose of 10^4^, 10^5^, 10^6^ EID_50_ in isolation units under biosafety level 4 conditions, and disease was monitored in all groups for 11 days for the appearance of clinical signs, weight gain, feed intake and mortalities in all groups. Birds were monitored for the presence of clinical sign and symptoms twice daily which were recorded as clinical scores 1 (no clinical signs), 2 (mild clinical signs), 3 (severe clinical signs), or 4 (dead/mortalities), as described previously (20, 21). At day 11 post-infection, the remaining animals were humanly euthanized.

#### Experiment 2: transgenic chickens and virus challenge

A total of 11 RCASBP(A)-chIFIT5 transgenic chicks (chIFIT5-HPAIV/vNDV +ve), 20 mock-inoculated chicks (mock-HPAIV/vNDV +ve) were either challenged with 10^4^ EID_50_ HPAIV or 10^5^ EID_50_ vNDV (clinical doses) on 12 days of age (1st day virus infection). Correspondingly, 10 chicks were kept as a naïve control group that were neither inoculated with retroviruses nor challenged with HPAIV or vNDV (mock-HPAIV/vNDV –ve). Before second challenge, a naïve group (Naïve-HPAIV/vNDV +ve) containing 4 chicks for HPAIV group and 3 chicks for NDV group was introduced. Except mock-HPAIV/vNDV –ve, individual birds in all groups were challenged with 10^6^ EID_50_ of HPAIV and vNDV. Disease was monitored for next 10 days for the appearance of clinical signs, weight gain, feed intake and mortalities in all groups. Birds were monitored for the presence of symptoms twice daily, and clinical signs were recorded as clinical scores 1 (no clinical signs), 2 (mild clinical signs), 3 (severe clinical signs), 4 (dead), as was described previously (20). Three birds from chIFIT5-HPAIV/vNDV +ve and two chicks from mock-HPAIV/vNDV +ve were sacrificed for comparison between transgenic and wild type birds. The experiment was terminated at day 17 post-challenge and all remaining animals were euthanized.

### Confirmation of transgene expression

Total RNA was extracted from trachea, which were collected from transgenic and non-transgenic chickens using TRIzol reagents (Invitrogen, Carlsbad, CA, USA). A total of 200 ng of RNA was used in PCR reactions using SuperScript® III Platinum® SYBR® Green One-Step qRT-PCR Kit (Invitrogen, Carlsbad, CA, USA) as we demonstrated earlier (5). The abundance of specific chIFIT5 mRNA was compared to the 28S rRNA in the Applied Biosystems Prism 7500 system. The reaction was carried out using the thermo profile reported earlier (5).

### Expression of innate immune genes

In order to determine the expression of innate immune genes, total RNA was extracted as described above using TRIzol reagents (Invitrogen, Carlsbad, CA, USA). A total of 200 ng of RNA was used in PCR reactions using SuperScript® III Platinum® SYBR® Green One-Step qRT-PCR Kit (Invitrogen, Carlsbad, CA, USA). The abundances of specific innate immune gene mRNA in trachea from transgenic (*n* = 05) and non-transgenic (*n* = 05) from both virus (HPAIV and vNDV) and mock-infected birds were compared to corresponding 28S rRNA and the average fold changes were determined (Supplementary Table 1). The reaction was carried out in ABI 7500 light cycler using the thermo profile described earlier (5). Primers for innate immune genes are provided in Supplementary Material.

### Processing of swab samples

Cloacal and oropharyngeal swabs were collected in 1 mL of 15% BHIB with antibiotics (10,000 IU/mL Penicillin G + 100 μg/mL Gentamycin + 20 μg/mL Amphotericin B) and were kept on ice, and then filtered through a 0.2 μm filter. The filtered material was stored at −80°C until all samples were collected, and then were subjected to HA as described previously (22).

### Histopathology

Selected tissues including trachea, brain, spleen, kidney and liver were collected and fixed by immersion in 10% neutral buffered formalin at room temperature for 48 h followed by processing and embedding in paraffin wax. Tissue sections of 5 μm were stained with Haematoxylin & Eosin and examined for microscopic lesions under light microscope.

### Statistical analysis

Pairwise comparisons of treated and control groups were performed using Student's *t*-test. Kaplan-Meier analysis was performed to calculate the survival rates. All statistical tests were conducted in the GraphPad Prism 7 (GraphPad Software, La Jolla, CA, USA).

### Ethics statement

All animal studies and procedures were carried out in strict accordance with the guidance and regulations of European and United Kingdom Home Office regulations. As part of this process, the work has undergone scrutiny and approval by the Animal Welfare and Ethical Review Body (AWERB) at The Pirbright Institute, UK.

### Results

#### Effective and constitutive expression of transgene using RCAS vector system

**Figure 1:**
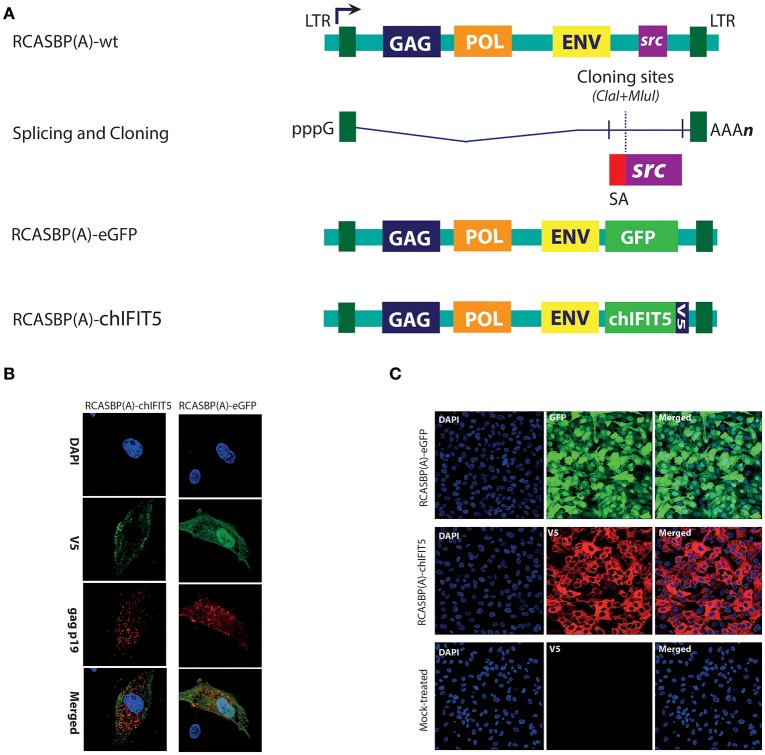
Generation and rescue of recombinant retroviruses expressing marker gene (GFP), and chIFIT5. **(A)** A schema for the generation of recombinant RCAS viruses in which *src* gene was replaced with either GFP, or chIFIT5. **(B)** Retroviruses were rescued in DF-1 cells and stained for retroviral structural gag protein and V5-taged fused to the chIFIT5, indicating the specific replication-competency of these retroviruses. **(C)** Immunofluorescence staining of the transgene in chicken fibroblasts expanded from individual clones expressing RCAS-mediated GFP and IFIT5 indicating stable and substantial expression of these proteins.

We have recently demonstrated that chicken IFIT5 is a crucial host restriction factor and can effectively subvert the replication of negative sense single stranded RNA viruses *in vitro* and *in ovo* (5). We next sought to investigate the potential of this antiviral protein in interfering virus replication *in vivo* and to propose a genetic marker of resistance against RNA viruses in poultry. For this purpose, efforts were made to generate transgenic chickens stably expressing chIFIT5, and to determine the antiviral potential of chIFIT5. For this purpose, we applied avian retroviruses (avian leukosis virus, ALV) vector-based expression system to assess impact of chIFIT5 gene against virus challenges in developing chicks. Specifically, the replication-competent and avian-origin RCAS (replication competent ALV long terminal repeats with a splice acceptor) system was applied to generate mosaic transgenic chicken (20, 23). The transgenes (GFP and chIFIT5) were expressed by the splice acceptor (SA) signal of the *src* viral oncogene, which were inserted between the unique polylinker sites as depicted in Figure 1A.

Initially, a reporter virus construct (RCASBP(A)-eGFP) was generated to monitor *in vitro* rescuing and progression of the virus. In addition, we constructed RCASBP(A)-chIFIT5 recombinant virus to generate mosaic-transgenic chicken embryos that are constitutively expressing chIFIT5. Both viruses were rescued in chicken embryo fibroblasts (DF-1) and replication was assessed by monitoring viral structural protein (gag) and transgene; GFP and V5-tagged chIFIT5, simultaneously (Figure 1B). Cell clones expressing RCAS-mediated GFP and chIFIT5 were individually and clonally expanded to obtain require stock density for transgenic embryo generation. Immunofluorescence staining of the transgene in chicken fibroblasts (Figure 1C) indicate stable and substantial expression of these proteins. These validated infectious chicken fibroblasts were used to generate transgenic chicken embryos.

#### Clinical and lethal dose assessment for HPAIV and vNDV

**Figure 2:**
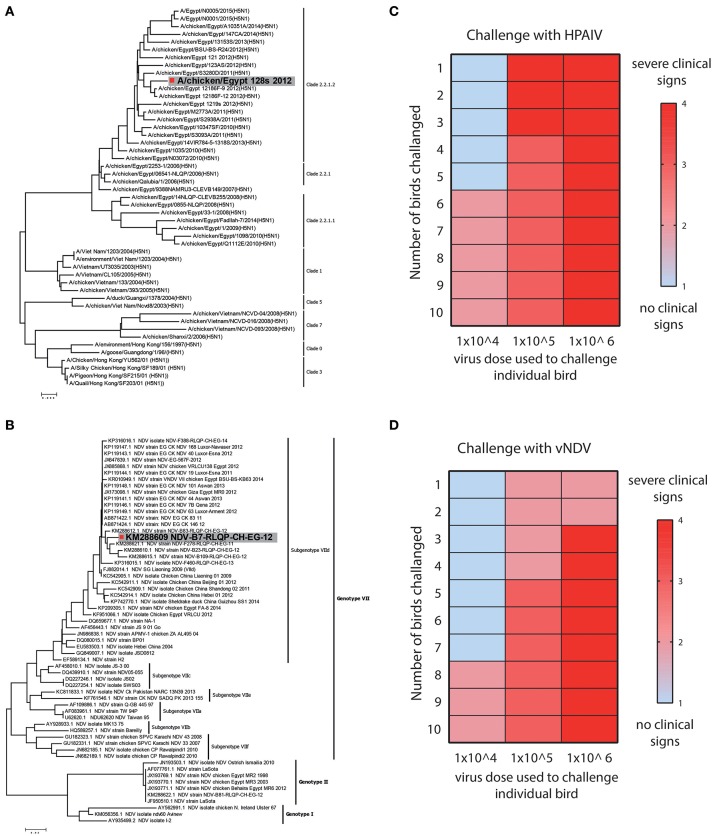
Strain selection and clinical and lethal doses assessment for HPAIV and vNDV. **(A)** Bioinformatics analysis of representative strains of H5N1 and **(B)** velogenic strains of NDV. Strains used in this study are colored gray. **(C)** Twelve days old SPF-chickens were challenged with the EID_50_ of 10^4^, 10^5^, and 10^6^ HPAIV or **(D)** vNDV and the disease was monitored until 11 days post-infection. Color gradient represent the severity of clinical signs.

HPAIV and vNDV strains cause severe infections and the clinical outcome of infection depends upon several factors including nature of the virus and genetics of the host (24). Based on the surface glycoprotein genes (haemagglutinin (HA) and fusion (F) genes), influenza and NDV can be divided into different subtypes, pathotypes, clades (Figure 2A) and genotypes (Figure 2B), respectively. Viruses belonging to H5-subtype that possess polybasic residues at the hemagglutinin protein cleavage site are reported to be highly pathogenic for both chickens and human (15), whereas Genotype VII strains of NDV are most prevalent and one of the major pathogen for clinical ND infections in chickens, around the globe (14).

Owing to direct correlation of virus dose with the severity of the clinical infections, we first determined the titre of HPAIV and vNDV inoculum, which induce clinical disease in chickens. Groups of ten SPF-chickens (12 days old) were challenged with different dosses (10^4^, 10^5^, or 10^6^ EID_50_) of HPAIV or vNDV and clinical disease scores were recorded until 11 days post-infection. Based on the level of disease severity, 10^4^ EID_50_ inoculum of H5N1 HPAIV induced clinical signs and was therefore considered as clinical dose. However, rapid death (lethal dose) was observed in birds inoculated with 10^6^ EID_50_ dose of H5N1 HPAIV (Figure 2C). For vNDV, most clinical signs were observed when 10^5^ EID_50_ virus particles (clinical dose) were used whereas a substantial mortality was observed in chickens, which were inoculated with 10^6^ EID_50_ virus dose (lethal dose) (Figure 2D). Prototype strains of H5N1 clade 2.2.1.2 (A/chicken/Egypt_128s_2012) and vNDV genotype VIId (NDV-B7-RLQP-CH-EG-12) with optimized doses 10^4^ EID_50_ and 10^5^ EID_50_ were used as inoculum to challenge transgenic chickens to demonstrate antiviral potential of IFIT5.

#### Improved survivability of transgenic chickens expressing chIFIT5 following infection with HPAIV and vNDV

**Figure 3:**
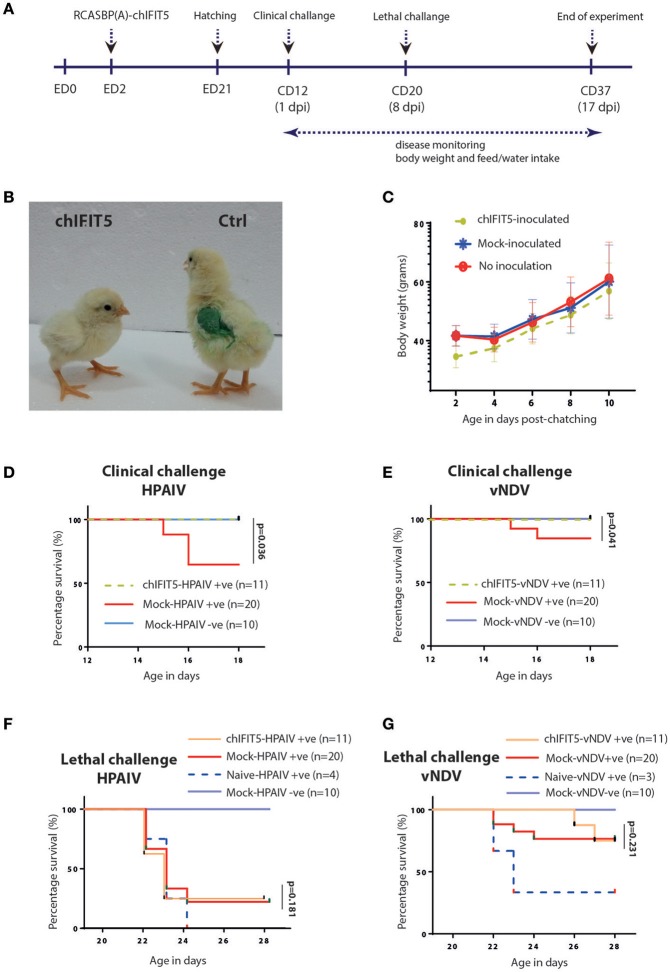
Generation of transgenic chickens and impact of chIFIT5 on physiological parameters of chicken. **(A)** Schema representing the time of transgenesis and challenge experiment. **(B)** Comparison of the body mass of chIFIT5 expressing transgenic chicken and control chicken. **(C)** Wait gain in transgenic and control chickens post-hatching and pre-challenge. **(D)** Survival curve of chickens against clinical dose of HPAIV, **(E)** vNDV, **(F)** lethal dose of HPAIV and **(G)** vNDV.

Mosaic transgenic chickens were generated by inoculating 2-day-old embryonated SPF eggs with recombinant RCAS virus-infected CEF expressing chIFIT5 (as shown in Figure 1C) or were inoculated with non-infectious CEF. Chicks hatched from infected embryonated eggs were reared in isolators until 12 days of age before challenge with clinical dose of HPAIV or vNDV at 12 days of age followed by lethal dose on 20 days of chick's age (8 days post first infection) (Figure 3A). Onset of clinical disease and health parameters were assessed until the end of experiment in IFIT5-expressing virus-challenged (chIFIT5-HPAIV/vNDV +ve), mock-inoculated and virus-challenged (mock-HPAIV/vNDV +ve) and mock-inoculated and non-virus-challenged groups (mock-HPAIV/vNDV –ve). Additionally, in order to delineate priming effect of clinical dose of the virus on the lethal dose, a naïve SPF group of chicken was included and challenged with lethal dose only.

While no detrimental effect of the chIFIT5 expression was observed on the embryonic development, hatchability of RCAS-chIFIT5 transgenic eggs was compromised compared to mock groups in two independently performed experiments. A total of 3 manually hatched and 6 naturally hatched chicks in RCAS-chIFIT5 group were weak and unhealthy, unable to drink, dehydrated and shown visceral gout, and all were humanly euthanized due to welfare reasons before the clinical challenge (12 days of hatching). No further macroscopic lesions or pathologies were noticed on necropsy investigation of these euthanized birds (data not shown). Interestingly, all RCAS-chIFIT5 transgenic chicks were hatched with substantially reduced body weight (Figure 3B). Except chicks (*n* = 6) that were died at 2nd, 7th or 9th day of hatching, RCAS-chIFIT5 transgenic chicks progressively regained the body weight and shown comparable weight with mock controls before first virus challenge at day 12 post hatch (Figure 3C).

A total of 30% mock-inoculated chicks were succumbed to HPAIV infection within 4 days of infection. However, RCAS-chIFIT5 transgenic chicks were fully protected with the clinical dose of HPAIV (chIFIT5-HPAIV +ve) as was observed in negative control (Mock-HPAIV –ve) (Figure 3D). Correspondingly, overexpression of IFIT5 protected all birds whereas 90% mock-inoculated chickens showed no apparent clinical disease when inoculated with clinical dose of vNDV (Figure 3E). In comparison, the group challenged with lethal dose of HPAIV showed rapid and sever disease signs and were humanly euthanized. Suggesting lethal dose of HPAI override endogenous levels of host innate responses including overexpression of IFIT5 (Figure 3F). Albeit corresponding mortality was observed in chicks challenged with lethal dose of vNDV, the lethality of the virus was meliorated for 4 days in RCAS-chIFIT5 transgenic chicks (Figure 3G). In order to exclude the possibility of priming effect of clinical dose on the protective efficacy of IFIT5 against lethal dose, naïve chicks of same age were challenged with corresponding lethal doses of HPAIV and vNDV (Naïve-HPAIV/vNDV +ve). As expected, 100% HPIAV and 66% vNDV challenged naïve chicks showed sever clinical signs and were culled or suddenly died due to infection within three days of challenge (Figures 3F,G). Taken together, the results demonstrate the functional role of IFIT5 to progressively protect chicks from clinical doses of both RNA viruses; however, overexpression of IFIT5 is insufficient as a stand-alone antiviral host factor that could completely protect chickens from the lethal dose of HPAIV and vNDV.

#### Transgenic chickens expressing chIFIT5 showed protection from clinical disease signs when challenged with sub-lethal dose of HPAIV and vNDV

**Figure 4:**
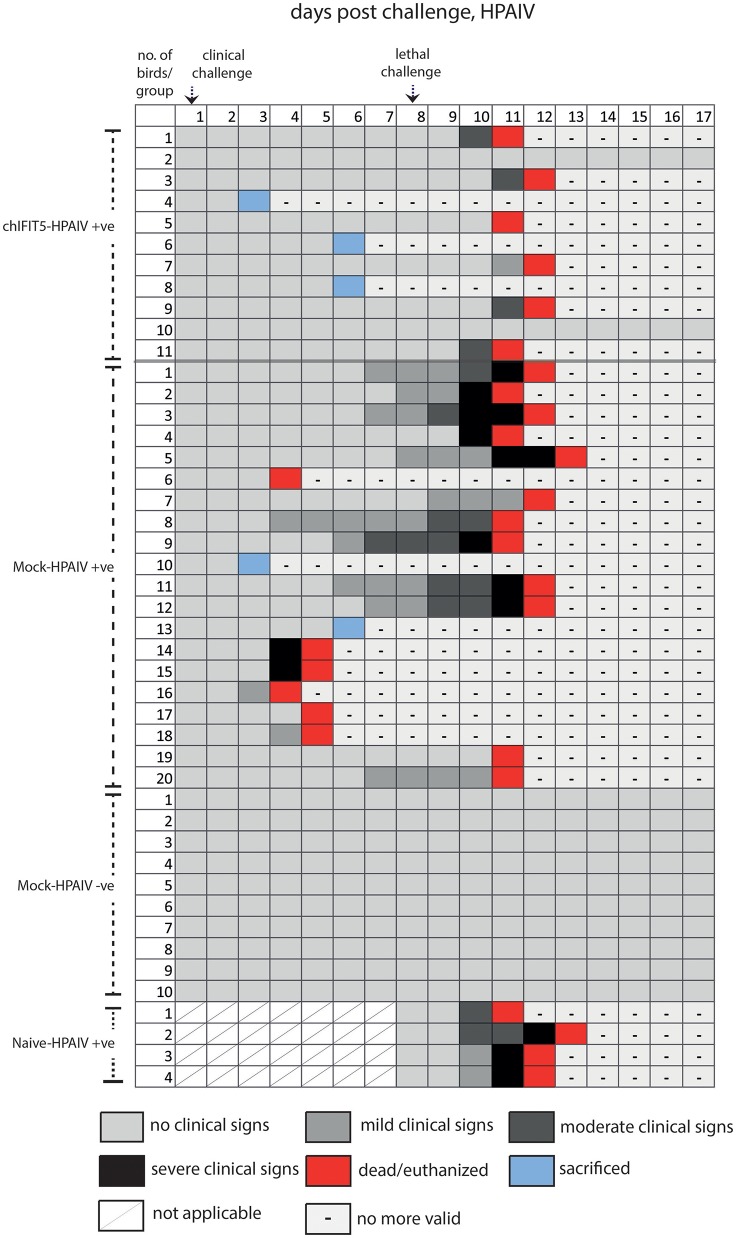
Clinical signs of individual chicken in different groups pre- and post-clinical and lethal challenge induced by the HPAIV.

**Figure 5:**
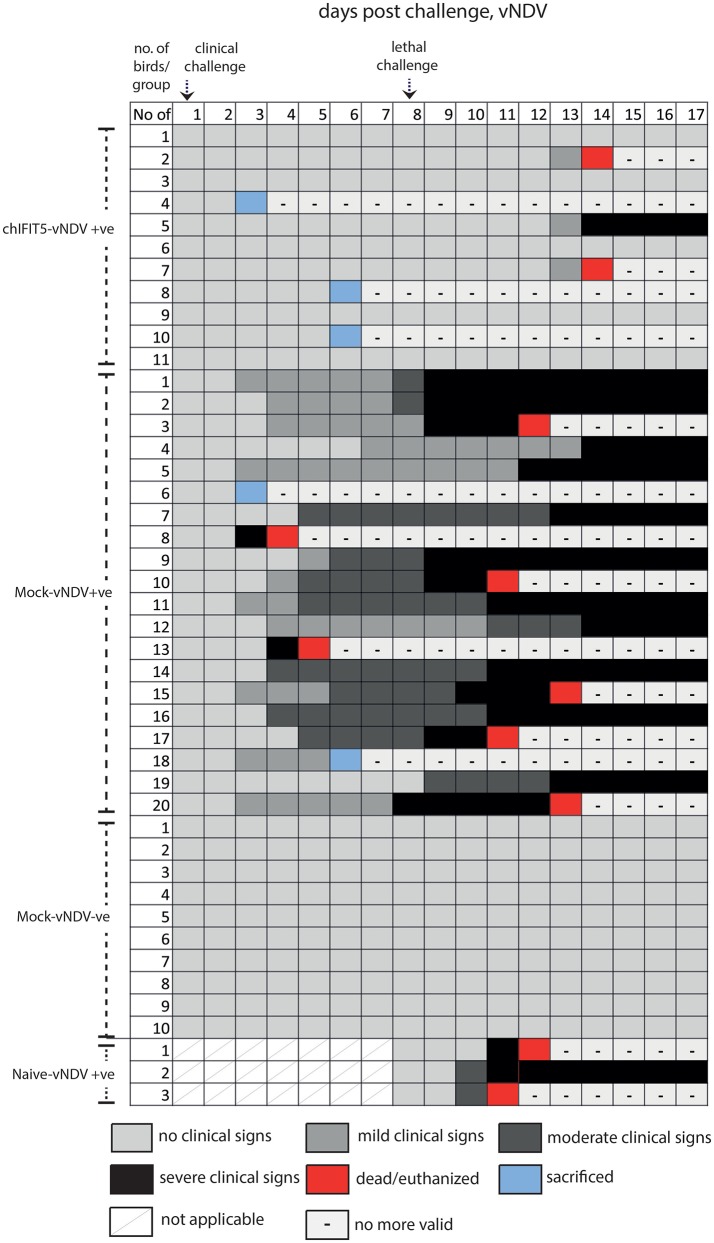
Clinical signs of individual chicken in different groups pre- and post-clinical and lethal challenge induced by the vNDV.

Intriguingly, IFIT5 fully protected transgenic chicks from morbid clinical signs when exposed to sub-lethal dose (10^4^ EID_50_) of HPAIV (Figure 4). In contrast, mock group (Mock-HPAIV +ve) showed sever clinical signs as early as third day following inoculation with clinical virus dose (10^4^ EID_50_). The clinical signs were further exacerbated when birds were follow-on exposed to lethal dose of HPAIV. While chicks in negative control (Mock-HPAIV –ve) remained healthy and corresponding challenged group shown the most clinical signs, overexpression of IFIT5 has substantially reduced the appearance of the clinical outcomes of HPAIV infections. These effects were markedly observed in vNDV infected RCAS-chIFIT5 transgenic chicks. Not only that IFIT5 expressing chicks were protected from the clinical challenge but substantially from the lethal challenge (Figure 4). Importantly, the clinical signs, which appeared in the RCAS-chIFIT5 transgenic chicks, were delayed by at least by 10 days (Figure 5). These results highlight the possible roles of IFIT5 in meliorating the clinical outcome of two highly pathogenic viruses in the poultry, influenza and NDV.

#### Confirmation of transgene expression and reduced virus shedding in IFIT5-expressing transgenic chickens challenged with HPAIV and vNDV

**Figure 6:**
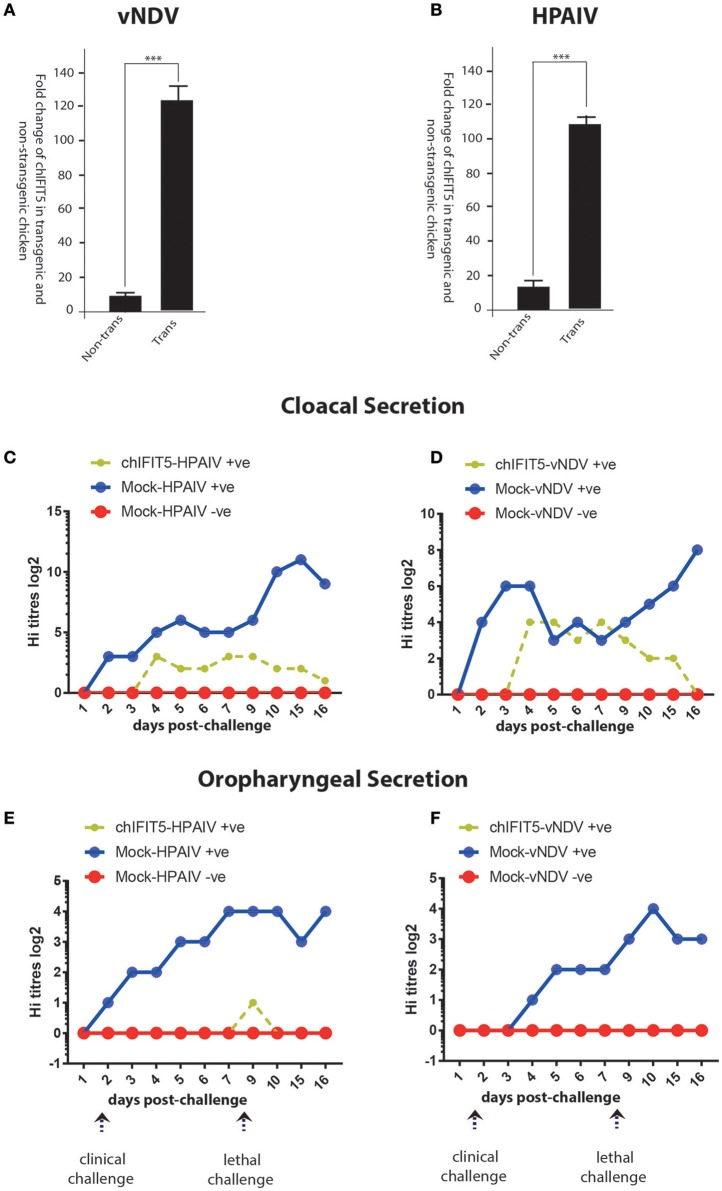
Expression of the transgene (chIFIT5), in vNDV-infected transgenic **(A)** and HPAIV-infected transgenic **(B)**, compared to non-transgenic chicken. Quantitative assessment of virus shedding in cloacal section for the **(C)** vNDV, **(D)** HPAIV and in oropharyngeal secretion for the **(E)** vNDV and **(F)** HPAIV in transgenic chickens expressing chIFIT5-challenged with virus (chIFIT5-vNDV/HPAIV +ve), mock-transgenic-virus challenged (mock-vNDV/HPAIV+ve) and mock control (mock-vNDV/HPAIV –ve). Days of clinical and lethal challenges are marked with arrows. *** indicate the level of significance at *p* > 0.05.

In order to demonstrate the successful expression of chIFIT5 in transgenic chickens, we exploited the codon-optimized version of RCAS-mediated chIFIT5 gene (5). A quantitative PCR that specifically detect codon-optimized chIFIT5 was established to demonstrate the expression of chIFIT5 as transgene and to differentiate the transgene from endogenously expressed chIFIT5. Using this assay, we detected a significantly higher level of IFIT5 in tracheal RNA collected from transgenic chicken compared to corresponding non-transgenic RNA. Relative to HPAIV-infected transgenic chicken (Figure 6A), the expression of chIFIT5 was significantly higher in vNDV-infected transgenic chickens (Figure 6B). Collectively, these data demonstrate the successful generation of transgenic chicken and the expression of transgene.

We next assessed if chIFIT5-mediated reduction in the clinical picture reflect upon the virus shedding in two most common routes of virus transmission and shedding; oral and cloacal routes. For this purpose, both cloacal and oropharyngeal swabs were collected from all three groups (chIFIT5-vNDV+ve, and mock-vNDV +ve, and mock-vNDV –ve) before the challenge and every day post-clinical and post-lethal dose challenges. Assessment of virus titres in the swab samples indicated a substantial shedding of both vNDV and HPAIV in cloacal secretions (Figures 6C,D). However, transgenic chickens, constitutively expressing chIFIT5 showed substantial reduction in both shedding titres (NDV: 8 log2 vs. 4 log2 in control, HPIAV; 3 log2 vs. 12 log2 in control) and the duration of shedding period following challenge with vNDV and HPAIV. In comparison to vNDV challenged chickens, the virus shedding was markedly reduced in cloacal swabs of HPAIV-challenged chicks.

Since shedding of both under-study viruses reported by the respiratory route of the chicken (22), we next assessed the magnitude of virus shedding in oropharyngeal swabs. Virus quantification analysis indicated secretion of both viruses (vNDV and HPAIV) in mock-transgenic and viruses-infected animals. However, intriguingly, the shedding of the viruses was fully blocked in chickens, which were engineered to constitutively express chIFIT5 in both clinical and lethal doses challenge (Figures 6E,F). A relatively low surge in virus shedding (1 log2) was observed in chickens that were challenged with lethal dose of HPAIV, which was subsided in 2 days. Collectively, these finding suggest that chIFIT5 play decisive roles in virus replication especially in the respiratory epithelial cells resulting in lower shedding levels in of viruses in the buccal cavity.

#### The ChIFIT5-expressing transgenic chickens show improved virus-induced microscopic pathologies and these are not associated with enhanced innate immunity

**Figure 7:**
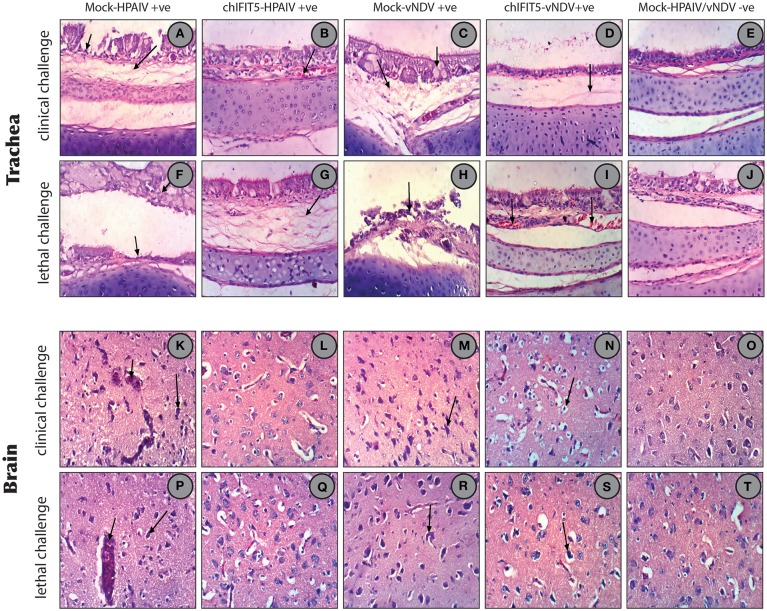
**(A)** Trachea of chicken infected with HPAIV (clinical challenged) showing focal necrosis of lamina epithelialis (small arrow) and oedema in the lamina propria/submucosa layer (large arrow). **(B)** Trachea of chicken expressing chIFIT5 and infected with HPAIV showing slight congested blood vessel. **(C)** Trachea of chicken infected with vNDV (clinical challenged) showing activation of mucous secreting glands (small arrow) and oedema in the lamina propria/submucosa layers (large arrow). **(D)** Trachea of chicken expressing chIFIT5 and infected with vNDV showing slight oedema in the lamina propria/submucosa layers. **(E)** Trachea of control chicken showing the normal histological layers. **(F)** Trachea of chicken infected with HPAIV (lethal challenged) showing focal necrosis of the mucosa (small arrow) and accumulation of mucous exudate in the lumen (large arrow). **(G)** Trachea of chicken injected expressing chIFIT5 and infected with HPAIV (lethal challenge) showing oedema in the lamina propria/submucosa layers. **(H)** Trachea of chicken infected with vNDV (lethal challenge) showing necrosis of the mucosa (arrow). **(I)** Trachea of chicken expressing chIFIT5 and infected with vNDV (lethal challenge) showing congested blood vessels. **(J)** Trachea of control chicken showing the normal histological layers. **(K)** Brain of chicken expressing chIFIT5 and infected with HPAIV (clinical challenge) showing congestion of cerebral blood vessel (small arrow) and necrosis of neurons (large arrow). **(L)** Brain of chicken expressing chIFIT5 and infected with HPAIV (clinical challenged) showing no histopathological changes. **(M)** Brain of chicken expressing chIFIT5 and infected with HPAIV (clinical challenged) showing necrosis of neurons (arrow). **(N)** Brain of chicken expressing chIFIT5 and infected with vNDV (clinical challenge) showing cellular oedema. **(O)** Brain of control chicken showing no histopathological changes. (**P**) Brain of chicken expressing chIFIT5 and infected with HPAI (lethal challenge) showing congestion of cerebral blood vessel (small arrow) and necrosis of neurons (large arrow). **(Q)** Brain of chicken expressing chIFIT5 and infected with HPAIV (lethal challenged) showing no histopathological changes. **(R)** Brain of chicken expressing chIFIT5 and infected with vNDV (lethal challenged) showing necrosis of neurons and neuronophagia (arrow). **(S)** Brain of chicken expressing chIFIT5 and infected with vNDV (lethal challenged) showing necrosis of some neurons and neuronophagia. **(T)** Brain of control chicken showing no histopathological changes. (All images were stained with H&E and are imaged at x400).

In order to estimate the magnitude of histopathological changes induced by these pathogenic viruses and the level of protection afforded by the stably expressing chIFIT5, organs (trachea, brain, spleen, kidneys and liver) were collected and histopathologically examined both at the clinical and lethal doses and compared with non-treated and mock controls. As expected, examination of trachea showed marked pathological lesions in mocked-transgenic and HPAIV and vNDV-challenged chickens including focal necrosis of lamina epithelialis mucosa, oedema in the lamina propria and congestion of mucosal blood vessels (after clinical-challenge). Severe histopathological alterations were also noticed after lethal-challenge including focal necrosis of the mucosa and accumulation of mucous exudate in the tracheal lumen. On the other hand, trachea collected from chickens, which were transgenically expressing chIFIT5 and infected with HPAIV and vNDV showed no histopathological changes except slight congested blood vessels, slight edema in the lamina propria/submucosa layers (Figures 7A–T). Congestion of cerebral blood vessel, necrosis of neurons and neuronophagia were observed in brain collected from mocked-transgenic and vNDV and HPAIV-challenged chickens. In brain examined from chicken, which were transgenically expressing chIFIT5 and infected with vNDV and HPAIV, showed lesions of slight cellular oedema and necrosis of sporadic neurons (Figures 7A–T). Corresponding histopathological lesions were observed in spleen, kidneys and liver (Supplementary Figure 1). Taken together, these results demonstrate that constitutive expression of chIFIT5 protects different organs against virus replication, which collectively reflect upon virus shedding, ameliorated clinical outcome and improved health status.

Owing to association of IFIT5 mediated induction of innate immunity (10, 13), we next investigated whether the improved protection in transgenic chickens was mediated by the innate immunity. For this purpose, total RNA was extracted from trachea, because both viruses can cause respiratory signs, and expression levels of five innate immune genes were determined. These genes were chosen based on their expression dynamics against viruses, and existing assays (1). Direct comparison of expression of innate immune genes indicated non-significant differences between transgenic (over-expressing chIFIT5) and non-transgenic (mock expressing) (Supplementary Table 1). These differences were not only noticed in vNDV challenged birds, which show enhanced protection but also in HPAIV-challenged birds, that were susceptible to influenza infections. These results propose that chIFIT5-confered protection was not associated with the induced innate immune responses and may link to direct antiviral affect of chIFIT5 (5, 9).

## Discussion

Poultry production is crucial for economy and food security for growing global human population. While the productivity of poultry has increased significantly over the years through selective breeding and improved genetics, the threats imposed by emerging and re-emerging pathogens have increased significantly especially since the introduction of intensive poultry-raising systems (25). Among these pathogens, avian viruses are at the forefront of constraints to enhanced productivity including avian influenza virus, NDV and infectious bronchitis virus amongst others. Upon infection with viruses, the innate immune responses mainly mediated by IFN-regulated proteins establish profound antiviral state in the host, and defines the gravity clinical disease outcome and associated decrease in productivity and mortalities. Avian species, especially chicken, are unique in the nature and dynamics of innate immunity (1) and are known to play crucial roles in the evolution and emergence of influenza viruses and their potential to cause infections in human (26).

Interestingly, chickens are lacking essential components of innate immune system (e.g., RIG-I, IRF3, IRF9); they yet mount profound innate immune responses against virus infections (1). Efforts have been made to delineate underlying molecular factors that uniquely control the virus-mediated innate immune-induction and have provided mechanistic insights, which differ between avian and mammals. Further understanding the alternative means immune regulation and antiviral defenses in chicken could establish the foundation to control avian viral disease and the chicken-mediated emergence of zoonotic infections such as influenza viruses (26).

Recently, in an effort to explore the functional implication of IFIT proteins in chickens, we reported that chicken encodes single IFIT5 proteins compared to several in humans and mice. This highly virus- and IFN-inducible protein interacted with RNA carrying a triphosphate group on its 5′ terminus (ppp-RNA) (5). These structures are present in the native form of negative sense viral genomics RNA. It is hypothesized that the interaction of IFIT5 with the 5′-ppp containing viral RNA potentially interferes with the transcription and subsequent translation of viral proteins (5). These interferences cooperatively impact negatively on the replication kinetics of RNA viruses. Most of these studies we conducted either in the cell culture models or *in ovo*, highlighting the potential of chIFIT5 as essential host antiviral restriction factor. To further investigate the antiviral activity of IFIT5 *in vivo*, we generated mosaic transgenic chicken that stably and constitutively express chIFIT5. These chickens were used to test the antiviral impact of IFIT5 on two highly pathogenic viruses; HPAIV and vNDV.

The mosaic transgenic chickens overexpressing chIFIT5 provided strong evidences that this cytokine possesses profound antiviral activities *in vivo*. These antiviral actions were sufficient to fully protect chicken against dose of viruses that otherwise cause clinical disease signs in chickens. Since the virus load in the field condition varies and the exposure of chickens to environmental stress contributes to the virus-induce pathologies, we additionally assess the impact of chIFIT5 overexpression on the pre-determined lethal dose (an amount virus dose that cause server disease and mortality in chickens). While over expression of chIFIT5 alone seems insufficient in affording complete protection from morbidity and mortality when challenged with “lethal dose (10^5^-10^6^ EID_50_)” of viruses. Nevertheless, the clinical outcome was substantially ameliorated when transgenic chickens were challenged with “clinal dose 10^4^-10^5^ EID_50_.” Furthermore, the results clearly ruled out the possibility that pre-exposure with “clinical dose” may have induced adaptive immune responses masking the impact of “lethal dose” on clinical outcome. These evidences clearly highlight the potential of innate immune in conferring resistance against HPAIV and vNDV.

It is noteworthy to mention that the protective role of chIFIT5 was assessed against two highly virulent viruses; highly pathogenic IAV and velogenic NDV. Both can cause up to 100% mortality in infected flocks. Therefore, it is plausible to hypothesize that the chIFIT5-alleviated morbidity against these highly pathogenic viruses may propose relatively profound impacts against viruses that are relatively less virulent, cause only clinical disease and low mortality. Additionally, vaccination of chIFIT5-expressing transgenic chicken may provide additional resistance to viruses, which warrant future investigation.

The challenge to generate transgenic chickens was mitigated by the use of RCAS-based retroviral gene transfer system. Based on previous studies (20, 27) and our recent report (5), retroviral-mediated transgene expression has been proposed as a convenient, economical and less laboratory-intensive system. Our *in vitro* investigation and *in ovo* assessments further confirmed the expression of transgenes in developing embryos and chicks. Additionally, we used qPCR to specifically detect chIFIT5 in tracheal RNA which is not only demonstrating the successful transgenesis but also re-enforcing our previous finding (5) that dictate the predominant expression of RCAS-mediated gene delivery in epithelial-enriched organs. While the RCAS-vector system is an efficient approach, it is by no means substitute for the genome edited-based (TALEN or CRISPR) transgenesis. It is therefore feasible that the phenotypic effect of chIFIT5 as antiviral may be profound in knocked-in primordial germinal cells and CRISPR/Cas9-generated transgenic chickens.

Overexpression of chIFIT5 has not only alleviated the manifestation of clinical disease signs in HPAIV and vNDV infected chickens but also reduced the viruses-induced pathological lesions and virus shedding. Since RCAS-based retroviral gene transfer system is predominantly effective in organs that are rich in endothelial cells (20, 27), we reasoned the complete blockage of virus shedding in trachea. This substantially reduced virus shedding correlated with the improved tracheal tissue health, which may highlight the expression and functional importance of chIFIT5 in mucosal surfaces. However, further investigations are warranted to ensure the enriched expression of this host factor in tracheal lining and its subsequent impact on virus replication. While a reduced body weight in transgenic chicks at hatching was observed, hatched chicks regained the weight swiftly and obtained comparable sizes to non-transgenic chicks. It requires additional investigations to fully delineate the mechanisms of this retarded embryo development; there are feasibilities that transgenically over-expression of the IFIT5 may interfere physiological and developmental processes of the chick. A single IFIT5 in chicken, compared to four or more in mammals, may likely to be restricted with its biological activities or may propose unconventional functions, which require future investigations.

Since previous reports (10, 13) indicate that human IFIT5 can positively regulate innate immune responses and hence inhibits viruses, we mapped chicken innate immune genes (*n* = 5; Mx, IFN-β, Viperin, IFI35, ADAP2) in transgenic chickens that were over-expressing chIFIT5. A non-significant difference was observed between transgenic and non-transgenic chicken. These finding propose existence of additional mechanisms that dictate the antiviral actions of chIFIT5 such as interaction with 5'-RNA and sequestration of viral RNA (5, 9, 10). Moreover, due to missing of additional IFIT genes in chicken (5), it is also plausible that chicken IFIT5 might carry functional plasticity for antiviral activities compared to human IFIT5, which require further research to delineate these mechanisms.

Taken together, we characterized the function of chIFIT5 in chicken and systemically analyzed the functional rationale for antiviral activities of chIFIT5 against RNA viruses using transgenic animal model. These finding propose the potential of innate immune in conferring resistance in chicken against viruses and provide evidences to generate future virus-resistance transgenic chicken for food security and to hamper transmission of zoonotic viruses to human.

## Author contributions

MM conceived the project and wrote the manuscript. MM, MI, DS, MR, and HH designed experiments. MR, DS, and RE performed experiments. MM, MR, DS, RN, MI, and HH participated in data analyses and reviewed the manuscript.

### Conflict of interest statement

The authors declare that the research was conducted in the absence of any commercial or financial relationships that could be construed as a potential conflict of interest.

## Supplementary material

Supplementary Figure 1 Histopathological lesions in different organs collected from transgenic or non-stransgenic chickens and challenged or not with HPAIV or vNDV. Labeling on the X-axes indicates the treatment and on the Y-axes are levels of virus challenge (clinical or lethal). Please refer to figure 7 in the main manuscript for detailed descriptions on the lesions.

Supplementary Table 1 Expression of innate immune genes in transgenic and non-transgenic chicken infected with under-study viruses.
