# Supplementary figures and images for: Chickens Expressing IFIT5 Ameliorate Clinical Outcome and Pathology of Highly Pathogenic Avian Influenza and Velogenic Newcastle Disease Viruses
Source: Front Immunol. 2018 Sep 14;9:2025. doi: 10.3389/fimmu.2018.02025 (PMC6149294; doi:10.3389/fimmu.2018.02025)

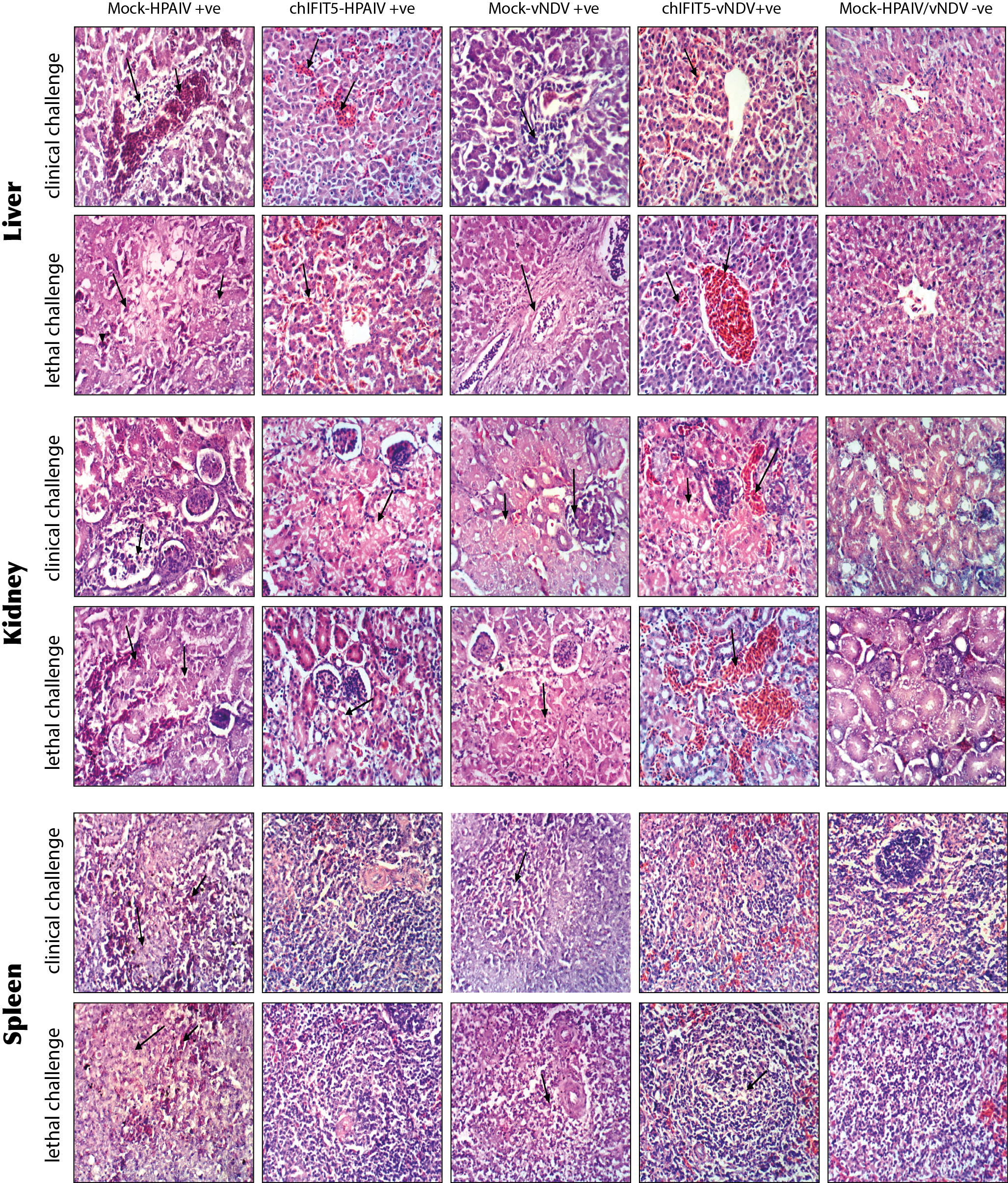

Supplement: Supplementary Figure 1 — Histopathological lesions in different organs collected from transgenic or non-stransgenic chickens and challenged or not with HPAIV or vNDV. Labeling on the X-axes indicates the treatment and on the Y-axes are levels of virus challenge (clinical or lethal). Please refer to figure 7 in the main manuscript for detailed descriptions on the lesions. [file Image_1.PNG]
