# Supplementary material for: Chickens Expressing IFIT5 Ameliorate Clinical Outcome and Pathology of Highly Pathogenic Avian Influenza and Velogenic Newcastle Disease Viruses
Source: Front Immunol. 2018 Sep 14;9:2025. doi: 10.3389/fimmu.2018.02025 (PMC6149294; doi:10.3389/fimmu.2018.02025)
Supplement: Supplementary Table 1 — Expression of innate immune genes in transgenic and non-transgenic chicken infected with under-study viruses. [file Table_1.DOCX]

**Supplementary Table**: Expression of innate immune genes in transgenic and non-transgenic chicken infected with viruses.

| **Gene Name** | **Forward & Reverse Primers** | **Fold change (HPAIV)*** | |  | **Fold change (vNDV)*** | | **Significance**  **(p value)**** |
| --- | --- | --- | --- | --- | --- | --- | --- |
|  |  | **Non-transgenic** | **Transgenic** | **Significance**  **(p value)**** | **Non-transgenic** | **Transgenic** |  |
| Mx | 5’CACTGCAACAAGCAAAGAAGGA3’  5’TGATCAACCCCACAAGGAAAA3’ | 89.0 | 91.0 | NS= 0.941 | 54.0 | 58.0 | NS= 0.896 |
| IFN-β | 5’CCTCCAACACCTCTTCAACATG3’  5’TGGCGTGCGGTCAAT3’ | 23.0 | 41.0 | NS= 0.582 | 21.0 | 18.0 | NS= 0.743 |
| Viperin | 5’TGCTTAAGGAGGCGGGAATG3’  5’CAGCTGGCCTACAAATTCGC3’ | 18.0 | 25.0 | NS= 0.721 | 27.0 | 31.0 | NS= 0.712 |
| IFI35 | 5’TGGTCCGCTATCCTCTGTCA3’  5’CTCGAGTGAGCCCAATCTCC3’ | 39.0 | 42.0 | NS= 0.798 | 48.0 | 54.0 | NS= 0.653 |
| ADAP2 | 5’CCCACTGGATGCATTTCCAC3’  5’AAAAAGTCTCTCGCTGGCGT3’ | 12.0 | 15.0 | NS= 0.822 | 18.0 | 13.0 | NS= 0.683 |
| 28S | 5’GGCGAAGCCAGAGGAAACT3’  5’GACGACCGATTTGCACGTC3’ | NA | NA | NA | NA | NA | NA |

*Fold change represents average of triplicate repeats run on five samples, compared to corresponding 28S and is rounded to full number. ** p values more than 0.05 were considered non-statistically significant (NS).
